# Luteolin Protects Against Noise‐Induced Hearing Loss via Mitigating Oxidative Stress and Apoptosis, With Potential Regulation of the EGR1/SPRY4 Axis

**DOI:** 10.1002/cns.70906

**Published:** 2026-05-01

**Authors:** Jia‐ning Guo, Hong‐kai Mei, Rui Liang, Peng‐wei Ma, Wei‐long Wang, Jia‐wei Chen, Zi Wang, Hao Yuan, Yu‐qiang Lun, Wei Gao, Lian‐jun Lu

**Affiliations:** ^1^ Department of Otolaryngology Head and Neck Surgery, Tangdu Hospital Air Force Medical University Xi'an China; ^2^ Department of Otolaryngology Head and Neck Surgery The Affiliated Suzhou Hospital of Nanjing Medical University, Suzhou Municipal Hospital, Gusu School, Nanjing Medical University Suzhou China

**Keywords:** apoptosis, EGR1, Luteolin, noise‐induced hearing loss, oxidative stress, SPRY4

## Abstract

**Aims:**

Noise‐induced hearing loss (NIHL) is a globally prevalent disorder caused by oxidative stress‐mediated hair cell death, with no effective clinical treatments. This study explored the protective effect of luteolin (LL), a natural antioxidant flavonoid, against NIHL and its underlying molecular mechanism.

**Methods:**

In vivo, mice received intratympanic LL injections around noise exposure, followed by ABR testing and cochlear immunofluorescence staining. In vitro, cochlear explants and HEI‐OC1 cells were pretreated with LL, followed by the induction of oxidative stress using tert‐butyl hydroperoxide (TBHP). Cellular viability, oxidative stress, and apoptosis were assessed. CRISPR/Cas9 technique was used to establish an Early growth response 1 (EGR1) knockout cell line. ChIP‐PCR and dual‐luciferase reporter assays clarified molecular mechanisms.

**Results:**

Intratympanic LL significantly attenuated noise‐induced auditory threshold elevation and outer hair cell loss in mice without affecting normal hearing. In vitro, LL dose‐dependently mitigated TBHP‐induced damage via regulating oxidative stress and apoptotic pathways, reversed TBHP‐induced EGR1 upregulation, EGR1 knockout enhanced oxidative stress resistance, and EGR1 directly regulated sprouty RTK signaling antagonist 4 (SPRY4) transcription, while LL inhibited TBHP‐induced SPRY4 upregulation.

**Conclusion:**

Luteolin protects against NIHL by alleviating oxidative stress and suppressing apoptosis, with potential involvement of the EGR1/SPRY4 signaling axis, representing a promising candidate for NIHL prevention.

## Graphical abstract

Luteolin attenuates noise‐induced auditory hair cell loss, as well as TBHP‐induced oxidative stress and apoptosis. EGR1 modulates the transcription of SPRY4; the expression levels of both factors are upregulated following TBHP exposure, and this inductive effect is abrogated by luteolin treatment.

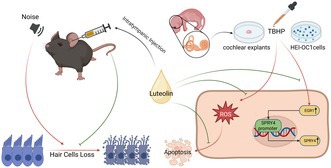

## 1 Introduction

Hearing loss is a major public health concern, with ~2.5 billion people projected to have auditory dysfunction by 2050 [1]. Statistics indicate that approximately 16% of adults' hearing loss stems from excessive noise exposure, ranking it as the second top etiological factor for sensorineural hearing loss—only after aging [2, 3, 4]. NIHL, caused by prolonged intense noise exposure [4, 5], primarily damages cochlear hair cells, and its prevalence has risen with worsening occupational and environmental noise pollution [6, 7].

The core pathological feature of NIHL is cochlear hair cell loss, whose underlying mechanism remains incompletely understood [8, 9, 10]. However, it is widely accepted that prolonged noise exposure triggers a sharp increase in energy consumption in the cochlea, thereby promoting the massive generation of ROS [11, 12]. This disrupts the oxidative stress balance in cochlear hair cells and ultimately initiates cell death [13, 14]. To further explore the pathogenesis of NIHL, in vitro models have been established using oxidants to induce oxidative stress and recapitulate noise‐induced injury. The most frequently used oxidants are hydrogen peroxide (H_2_O_2_) and TBHP. As an organic peroxide, TBHP is resistant to degradation by canonical antioxidant enzymes, readily penetrates cell membranes, and sustains continuous intracellular production of ROS, making it adopted for establishing in vitro models of NIHL [15, 16].

Numerous studies have focused on mitigating oxidative stress in hair cells via diverse approaches [12, 17]. These include the application of antioxidants such as N‐acetylcysteine, resveratrol, and D‐methionine [18, 19, 20]. While some of these agents have demonstrated efficacy in animal models, no drug has yet successfully passed clinical trials for NIHL. LL, a natural flavonoid extracted from various plants, possesses potent antioxidant and neuroprotective properties attributed to its 3′,4′‐catechol structure that scavenges free radicals and chelates redox‐active metals [21, 22]. LL has been demonstrated to safeguard the heart, kidney, and nervous system against damage induced by oxidative stress [23, 24, 25, 26]. To date, however, no published research has investigated whether LL can exert a protective effect against NIHL.

EGR1 is an immediate‐early transcription factor, rapidly activated by cellular stress without requiring de novo protein synthesis [27]. EGR1 modulates the transcription of numerous target genes, thereby playing important roles in cell survival, proliferation, and stress adaptation [28, 29, 30]. Recent studies implicate EGR1 in oxidative stress regulation; in ischemic cardiomyopathy and Parkinson's disease, EGR1 expression is significantly upregulated, which is accompanied by increased ROS production and elevated oxidative stress levels. In contrast, silencing or knocking out EGR1 can effectively alleviate oxidative stress, thereby mitigating tissue damage [31, 32]. However, the role of EGR1 in NIHL remains elusive.

SPRY4, a Sprouty family member, regulates embryonic development and tissue homeostasis and is linked to oxidative stress. In acute lung injury and polycystic ovary syndrome models, SPRY4 is upregulated; its knockout or knockdown attenuates oxidative stress and restores function [33, 34]. Although EGR1 is known to transcriptionally regulate SPRY1 and SPRY3 [35], its direct regulation of SPRY4 remains unclear. EGR1 can modulate fibroblast growth factor signaling [36], which induces SPRY4 expression [37], suggesting a potential indirect regulatory mechanism. The direct transcriptional relationship and the role of SPRY4 in NIHL require investigation.

We hypothesized that LL protects against NIHL by mitigating oxidative stress and suppressing apoptosis, potentially via regulating the EGR1/SPRY4 axis. We verified this hypothesis using in vivo (noise‐exposed mice) and in vitro (TBHP‐induced cochlear explants, HEI‐OC1 cell injury, and EGR1‐knockout HEI‐OC1 cells) models, elucidating NIHL pathogenesis and providing a theoretical basis for the clinical application of LL.

## 2 Methods and Materials

### 2.1 Animals

Male C57BL/6 mice aged 5–6 weeks were purchased from the university's Animal Experiment Center, acclimated for 1 week. Throughout the study, all the mice were housed in standard environmental settings (22°C ± 1°C, 12 h light/dark cycle) with unrestricted availability of laboratory chow and sterile water.

### 2.2 Auditory Brainstem Response (ABR) Testing

Pre‐exposure and 14 days post‐noise exposure, ABR assessments were performed. Mice were anesthetized with intraperitoneal 1% sodium pentobarbital (10 μL/g) and placed in a soundproof chamber. Subcutaneous electrodes were inserted at the test ear (reference), skull vertex (recording), and contralateral ear (ground). Short tones (8, 16, 32 kHz) were delivered via a TDT RZ6 system, with sound pressure levels ranging from 20 to 90 dB, decreased in 10 dB increments (5 dB increments if waveforms were unrecognizable). The ABR threshold was the minimum SPL eliciting stable wave II in three consecutive trials. Body temperature was maintained with a heating pad, and threshold determinations were performed by separate experimenters to minimize bias.

### 2.3 Drug Administration

LL (TargetMol, T1027) was administered via intratympanic injection. A 50 mg/mL stock solution (DMSO) was diluted 10‐fold in normal saline containing 20% sulfobutylether‐β‐cyclodextrin (ADAMAS‐BETA, 01377227), sterilized with a 0.22 μm filter, and stored until use. Mice were randomly assigned into four groups: (1) DMSO control (DMSO injection, no noise); (2) DMSO + noise (DMSO injection + noise exposure); (3) LL control (LL injection, no noise); and (4) LL + noise (LL injection + noise exposure). Two injections (5–10 μL) were given: 24 h pre‐noise and immediately post‐noise.

To minimize damage to the tympanic membrane during injection, the procedure was performed as previously described [38]. Mice were anesthetized, the left ear was positioned upward, and the external auditory canal was stabilized with a spring speculum. A 34G needle punctured the tympanic membrane, and 5–10 μL of working solution was slowly injected into the tympanic cavity. Mice were placed on a heating pad until anesthesia recovery (Figure S1). The right ear was left untreated as an internal control.

### 2.4 Noise Exposure

Noise exposure was conducted in a soundproof chamber. The mice were individually positioned in compartments of custom‐manufactured metal cages (dimensions: 15 cm × 5 cm × 5 cm). Four loudspeakers interfaced with the TDT RZ6 system were positioned inside the experimental chamber to deliver 2–20 kHz broadband noise at a sound pressure level (SPL) of 102–104 dB; this intensity was calibrated using a sound level meter placed within the cage compartments before the initiation of noise exposure. The noise exposure duration was 2 h; during this period, the mice were allowed free movement within their respective compartments but had no access to food or water.

### 2.5 Cochlear Surface Preparations

Two weeks post‐noise exposure, mice were sacrificed, and cochleae were dissected, cleaned of extraneous tissues, subjected to overnight fixation in 4% PFA at 4°C and 3‐day decalcification in 10% EDTA at 4°C, with the decalcifying medium changed daily. Basilar membranes were microdissected and transferred to 96‐well plates.

### 2.6 Cochlear Explants Culture

Cochlear explants were harvested from postnatal day 2 Sprague–Dawley (SD) rat pups. The cochlear explants were dissected in Hank's solution and spread flat on gel droplets. These gel droplets were composed of Basal Medium Eagle (BME, Sigma‐Aldrich, B9638) and Collagen I (CORNING, 354,249), and had been pre‐solidified at 32°C for 30 min. Explants were cultured in serum‐free medium (SFM) supplemented with BME, BSA (Sigma‐Aldrich, V900933), and Insulin‐Transferrin‐Selenium (Sigma‐Aldrich, I3146). After 4 h of adhesion, additional SFM was added.

Explants were divided into four groups: (1) Control group: no treatment; (2) LL group: treated with 50 μM LL for 4 h; (3) TBHP group: treated with 100 μM TBHP (Sigma‐Aldrich, 458,139) for 2 h; (4) LL + TBHP group: pretreated with 50 μM LL for 2 h and then treated with 100 μM TBHP for 2 h.

### 2.7 Immunofluorescence Staining

For immunofluorescence staining, cochlear surfaces or explants were permeabilized with 1% Triton X‐100 for 15 min, blocked with immunofluorescence blocking solution (LEAGENE, IH0338) for 1 h at room temperature, and incubated overnight at 4°C with primary antibodies: rabbit anti‐myosin VIIa (Proteus BioSciences, 25–6790, 1:500, hair cell marker) and mouse anti‐β‐tubulin 3/Tuj1 (GeneTex, GTX631836, 1:1000, nerve fiber marker). After PBS washes, the samples were incubated with Alexa Fluor 488‐conjugated goat anti‐rabbit IgG (Invitrogen, A‐11008, 1:1000), Alexa Fluor 594‐conjugated goat anti‐rabbit IgG (Invitrogen, A‐11012, 1:1000), and Alexa Fluor 594‐conjugated goat anti‐mouse IgG (Invitrogen, A‐11005, 1:1000)—fluorescent secondary antibodies—for 2 h at room temperature in the dark. DAPI staining (Solarbio, C0065) was performed for 15 min and samples were mounted with antifade medium thereafter. Images were captured using an Olympus FV3000 laser confocal microscope.

### 2.8 Cell Culture

HEI‐OC1 cells were cultured in DMEM with 10% FBS (Gibco, 10099141C) and 0.1 mg/mL ampicillin (Solarbio, A1170) under humidified conditions at 37°C with 5% CO_2_. Cells were inoculated in 96‐well plates (5000 cells/well), 6‐well plates (5 × 10^5^ cells/well), or 1 cm confocal dishes (2 × 10^5^ cells/dish) and cultured for 24 h to adhere.

### 2.9 Cell Viability Assay

CCK‐8 (PCM, PC‐1050) was used to assess cellular viability. After the completion of treatment, the cells were stained with medium containing 10% CCK‐8 reagent for 2 h, and absorbance at 450 nm was measured.

### 2.10 Cell Protein Extraction and Western Blot Analysis

RIPA lysis buffer (Beyotime, P0013B) containing PMSF (Beyotime, ST506) was used to lyse the cells. Protein samples were fractionated by SDS‐PAGE (Solarbio, P1200), transferred to PVDF membranes (Millipore, IPVH00010); the membranes were blocked with skim milk and incubated with primary antibodies at 4°C overnight: 4‐HNE (GeneTex, GTX17571, 1:1000), cleaved caspase‐3 (Cell Signaling Technology, 9664S, 1:1000), Bcl‐2 (GeneTex, GTX100064,1:1000), BAX (GeneTex, GTX109683, 1:1000), EGR1 (Invitrogen, MA5‐15008, 1:1000), SPRY4 (Biodragon, RM5688, 1:1000), and GAPDH (Invitrogen, MA5‐15738, 1:4000). Membranes were incubated with secondary antibodies (1 h, room temperature), and signals were detected via chemiluminescence.

### 2.11 Flow Cytometry

Following experimental treatment, the cells were collected and resuspended in the following staining solutionHEI‐OC1 cells were detached with trypsin, collected by centrifugation, and the resulting cell pellet was reconstituted in assay‐specific flow cytometry staining buffers for incubation:

Intracellular ROS detection: DCFH‐DA probe (YEASEN, 5010ES01) was added, followed by incubation at 37°C for 30 min.

Mitochondrial membrane potential assessment: JC‐1 staining solution (Beyotime, C2003S) was applied, followed by incubation at 37°C for 20 min.

Apoptosis analysis: Annexin V‐FITC/PI dual staining solution (Invitrogen, V13241) was added, followed by incubation at room temperature for 15 min.

Upon completion of staining, all samples were immediately analyzed by flow cytometer.

### 2.12 DHE Assay

DHE (Beyotime, S0063) was used to detect intracellular oxidative stress levels. After experimental treatment, the medium was aspirated, and the cells were washed with PBS. A 5 μM DHE working solution was added for incubation for 30 min at 37°C under light‐excluded conditions. Following staining, the cells were rinsed with PBS, and images were acquired immediately using a laser confocal microscope.

### 2.13 TUNEL Assay

In Situ Cell Death Detection Kit (Roche, 12,156,792,910) was used to perform TUNEL staining. Following treatment, the cells were fixed in 4% PFA and permeabilized using 0.1% Triton X‐100. The prepared TUNEL working solution was then applied, and the cells were incubated for 1 h at 37°C under light‐excluded conditions. Nuclear counterstaining was performed with DAPI, and fluorescence images were acquired using a laser scanning confocal microscope.

### 2.14 Construction of EGR1 Knockout Cell Lines

CRISPR/Cas9 technology was used to generate HEI‐OC1 cell lines with stable EGR1 gene knockout. Plasmid vectors encoding CRISPR/Cas9 components targeting the EGR1 gene were introduced into the cells using the K4 Transfection System (Biontex, RKMK4M1). At 24 h post‐transfection, transfected cells were subjected to selection with 2 μg/mL puromycin (Solarbio, P8230) for 5 days to isolate individual cell colonies. Monoclonal colonies were subsequently validated for EGR1 knockout efficiency via Western blot analysis. Specifically, EGR1 sgRNA primer sequences for recombinant plasmid construction are provided as follows (5′‐3′):

Forward: GTTGGGGTACTTGCGCATG.

Reverse: CATGCGCAAGTACCCCAAC.

### 2.15 ChIP‐PCR

SimpleChIP Enzymatic Chromatin IP Kit (Cell Signaling Technology, 9003S) was used to perform ChIP‐PCR experiments. TBHP‐treated cells were cross‐linked with 1% formaldehyde. DNA was enzymatically digested, and chromatin was obtained by sonication. Immunoprecipitation was performed with anti‐EGR1 antibody (Cell Signaling Technology, 4154S) and normal rabbit IgG (negative control, Cell Signaling Technology, 2729S). Immune complexes were adsorbed by magnetic beads, followed by elution, de‐crosslinking, and spin column DNA purification. The DNA was then quantified via quantitative real‐time PCR. The primers for the SPRY4 promoter region are provided as follows (5′‐3′):

Forward: CCTGTCCGCTGAATGGCTTCC.

Reverse: GTGACCAAGAGAAGGCAACAGAGC.

### 2.16 Dual‐Luciferase Reporter Gene Assay

Cells were co‐transfected with pGL‐4.10[luc2] vector harboring either the wild‐type (WT) or mutant (MUT) SPRY4 promoter (Table S1), the EGR1 overexpression plasmid (pCDNA3.1‐EGR1(mouse)‐3 × FLAG‐SV40‐Neo), and Renilla luciferase internal reference plasmid. After transfection, cells were lysed, and supernatants were mixed with firefly and Renilla luciferase substrates sequentially. Luciferase activity was measured using the Dual‐Luciferase Reporter Assay Kit (Beyotime, RG027).

### 2.17 Statistical Analysis

Statistical analyses were performed using GraphPad Prism 9.0. Unpaired *t*‐tests were used for two‐group comparisons, and one‐way ANOVA with Tukey's post hoc test for multi‐group comparisons. Significance was set at *p* < 0.05, and data are expressed as mean ± SD. Statistical symbols are defined as follows: *p < 0.05; **p < 0.01; ***p < 0.001; ****p < 0.0001; ns = not significant.

## 3 Results

### 3.1 LL Treatment Alleviates Noise‐Induced Auditory Threshold Shifts and OHC Loss

**FIGURE 1:**
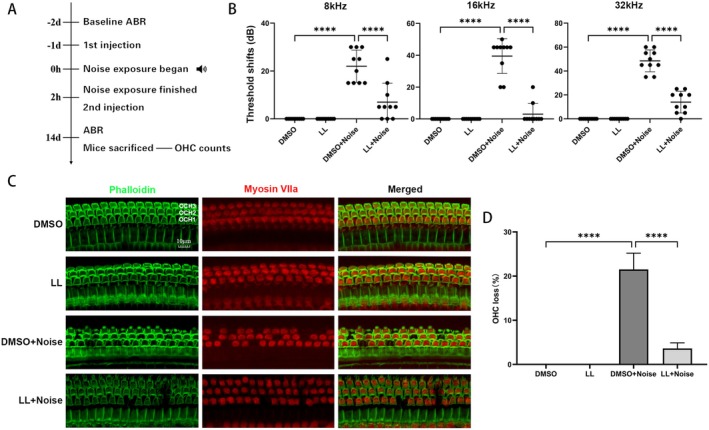
Intratympanic injection of LL attenuates the elevation of auditory thresholds and OHC depletion caused by noise exposure. (A) Experimental procedure. (B) LL significantly mitigates noise‐induced shifts in ABR threshold. *n* = 10. (C, D) Representative fluorescence images and quantitative analysis of hair cell loss rate show that LL treatment significantly alleviates noise‐induced OHC loss. Scale bar = 10 μm. *n* = 3. ****p < 0.0001.

To ascertain the capacity of LL to protect against NIHL, mice were treated as outlined in Figure 1A. ABR testing revealed that noise exposure caused a significant elevation in auditory thresholds at 8, 16, and 32 kHz in the DMSO + noise group compared versus controls. In contrast, the LL + noise group exhibited significantly lower threshold shifts at these frequencies compared with the DMSO + noise group (8 kHz: 7.0 ± 7.888 dB vs. 22.0 ± 6.749 dB; 16 kHz: 3.0 ± 6.749 dB vs. 39.5 ± 10.92 dB; 32 kHz: 14.0 ± 9.068 dB vs. 48.5 ± 9.144 dB, all *p* < 0.0001). Notably, no significant difference in ABR thresholds was observed between the DMSO control and LL control groups, confirming that intratympanic LL administration itself does not affect normal auditory function (Figure 1B).

After noise exposure, OHCs in the basal turn of the cochlea undergo degeneration and loss. To verify the protective effect of LL on cochlear hair cells after noise exposure, we performed immunofluorescence staining of cochlear surface preparations 14 days post‐noise exposure. Immunofluorescence revealed severe OHC loss in the basal turn after noise exposure, which was markedly attenuated by LL (Figure 1C,D).

### 3.2 LL Treatment Attenuates TBHP‐Induced Damage in Cochlear Explants

**FIGURE 2:**
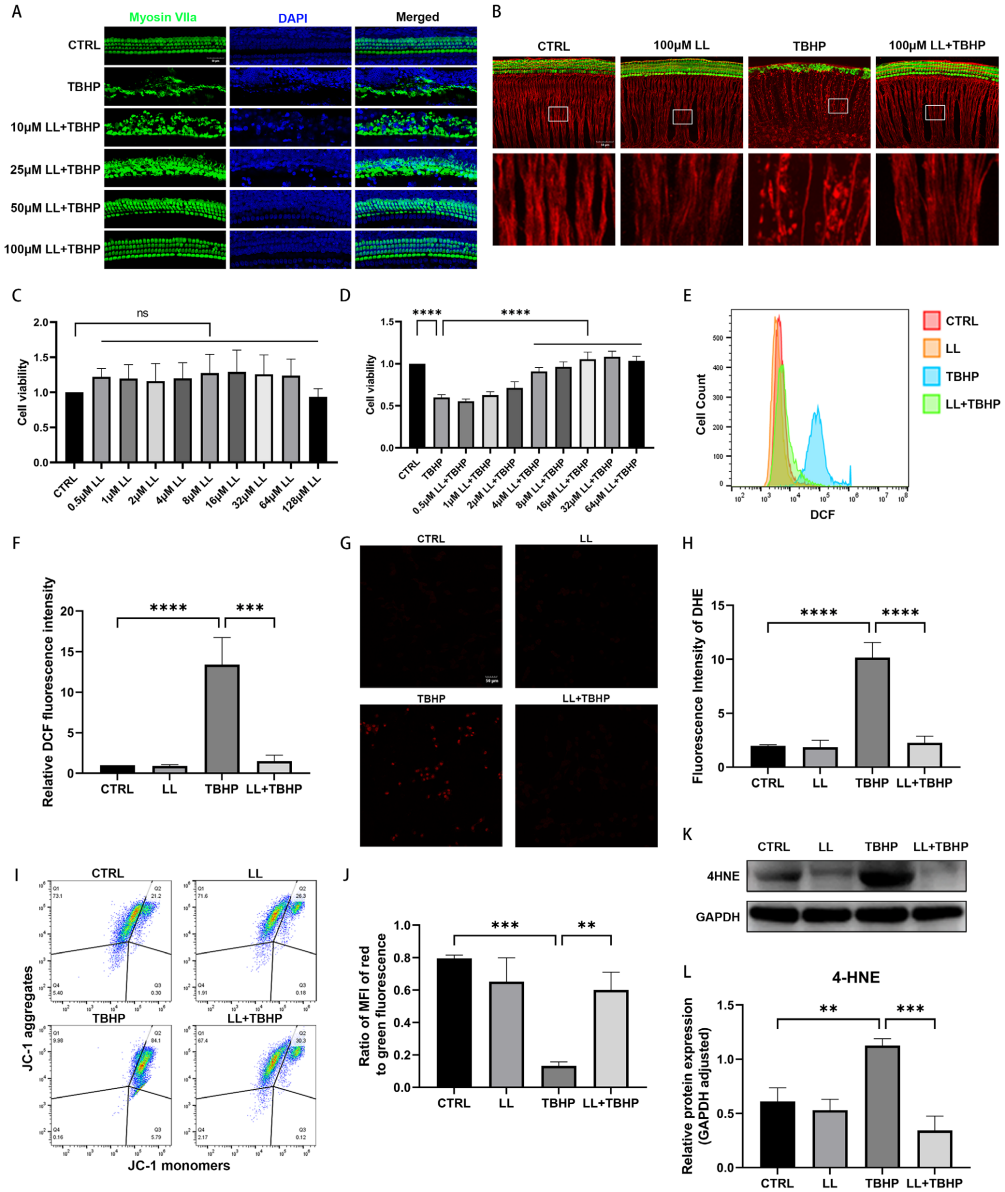
LL attenuates TBHP‐induced damage in cochlear explants and HEI‐OC1 cells. (A) Representative fluorescence images showing TBHP‐induced hair cell loss and the protective effects of pretreatment with different concentrations of LL. (B) Representative fluorescence images of cochlear explants and partial magnifications, with hair cells labeled by Myosin VIIa (green) and nerve fibers labeled by β‐Tubulin 3/Tuj1 (red). (C, D) Cell viability was measured using the CCK‐8 assay following treatment with LL and TBHP. (E, F) Representative flow cytometry images of DCFH‐DA and the relative median fluorescence intensity (MFI) of DCF. (G, H) Representative fluorescence images and quantification of DHE‐stained cells in different treatment groups. (I, J) Representative flow cytometry images of JC‐1 and ratio of MFI of red fluorescence to green fluorescence. (K, L) Representative Western blot images and semiquantitative gray value analysis of 4‐HNE levels. *n* = 3 for all experiments. Scale bar = 50 μm for all fluorescence images. **p < 0.01; ***p < 0.001; ****p < 0.0001; ns = not significant.

To further investigate the protective effect of LL on cochlear tissue, we used TBHP to induce oxidative damage in cochlear explants cultured in vitro. As illustrated in Figure 2A, TBHP exposure triggered significant hair cell depletion. In contrast, pretreatment with LL conferred a dose‐dependent protective effect against TBHP‐induced hair cell loss. Meanwhile, TBHP treatment led to prominent structural impairment of auditory nerve fibers in cochlear explants, as evidenced by the loss of nerve fibers arranged in parallel with hair cells and the rarefaction of longitudinal nerve fiber bundles. Notably, LL pretreatment preserved nerve fiber integrity and restored ordered structure (Figure 2B).

### 3.3 LL Treatment Mitigates TBHP‐Induced Cell Viability Reduction and Oxidative Stress Damage in HEI‐OC1 Cells

To explore the cytoprotective actions of LL on cochlear hair cells, we utilized the CCK‐8 assay to examine the viability of the HEI‐OC1 cell line. As shown in (Figure 2C,D), LL had no cytotoxicity at concentrations ranging from 0.5 to 128 μM and dose‐dependently improved cell viability in TBHP‐treated cells, with statistically significant effects starting at 4 μM.

To evaluate the extent of cellular oxidative stress, DCFH‐DA staining was performed, and the median fluorescence intensity (MFI) of DCF was quantified via flow cytometry. As shown in (Figure 2E,F), TBHP exposure induced a marked elevation in intracellular ROS levels. DHE staining further confirmed that TBHP treatment triggered a significant elevation in intracellular oxidative stress. Additionally, JC‐1 staining assays revealed that TBHP‐treated cells exhibited a marked reduction in mitochondrial membrane potential (Figure 2I,J). Importantly, LL pretreatment markedly reversed all these TBHP‐induced alterations.

4‐Hydroxynonenal (4‐HNE), a classic lipid peroxidation marker, was detected by Western blotting to reflect oxidative stress‐related protein modification. TBHP treatment significantly increased intracellular 4‐HNE levels, whereas LL pretreatment markedly attenuated this TBHP‐induced elevation (Figure 2K,L).

### 3.4 LL Treatment Alleviates TBHP‐Induced Apoptosis in HEI‐OC1 Cells

**FIGURE 3:**
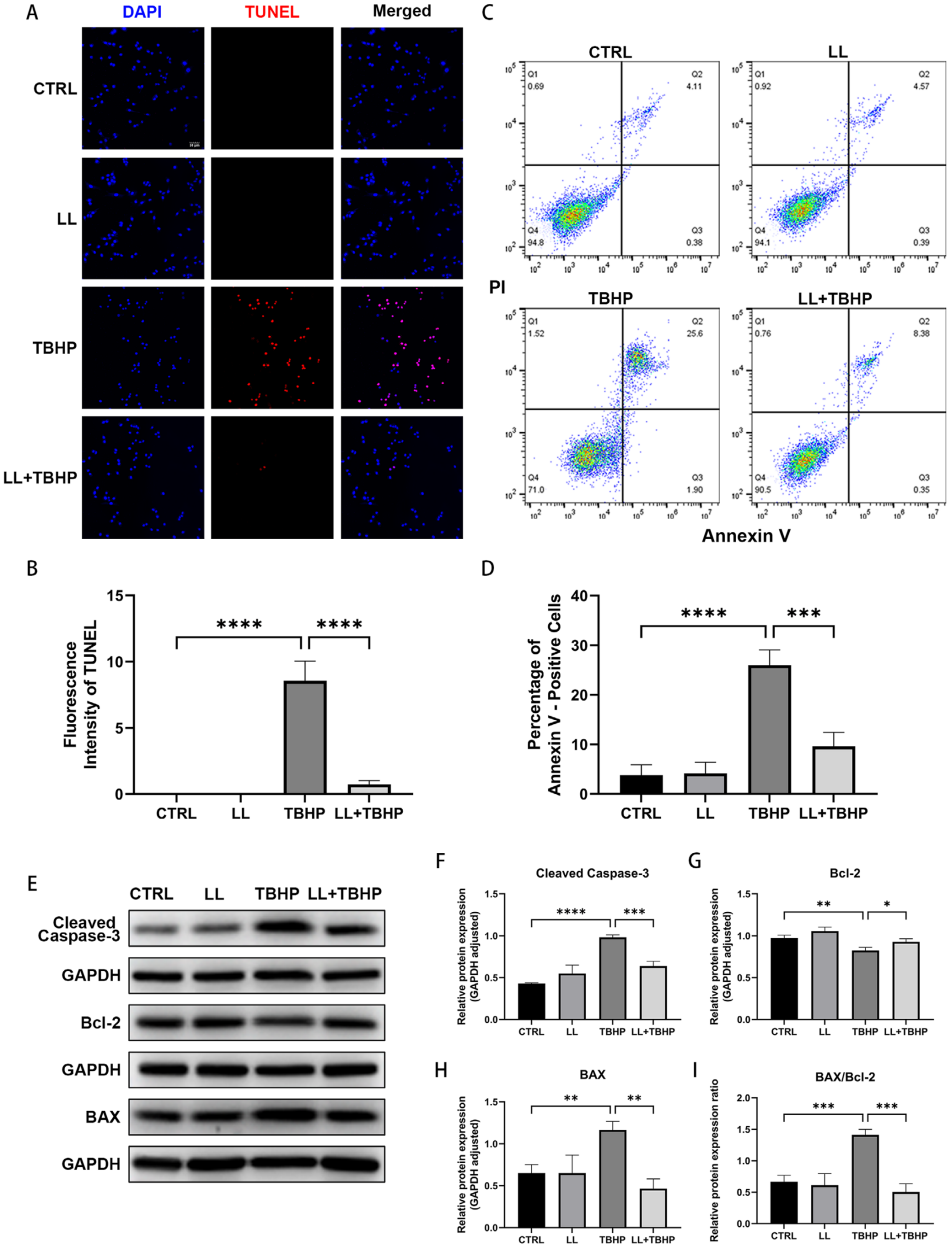
LL attenuates TBHP‐induced apoptosis in HEI‐OC1 cells. (A, B) Representative fluorescence images of DAPI/TUNEL co‐labeling and quantitative analysis of TUNEL‐positive cells following treatment with LL and TBHP. Scale bar = 50 μm. (C, D) Representative flow cytometry images of Annexin V‐FITC/PI staining and the quantification of apoptotic cells (Q2 + Q3 quadrants). (E–H) Representative Western blot images showing the protein expression levels of cleaved caspase‐3, Bcl‐2, and BAX, and semiquantitative gray value analysis. (I) The relative expression ratio of BAX to Bcl‐2. *n* = 3 for all experiments. *p < 0.05; **p < 0.01; ***p < 0.001; ****p < 0.0001.

To investigate LL's regulatory role in apoptotic pathways, we conducted TUNEL staining and Annexin V‐FITC/PI flow cytometric assays. TBHP stimulation significantly increased the number of TUNEL‐positive cells and markedly elevated the apoptotic rate, whereas LL pretreatment effectively reversed these effects (Figure 3A–D).

We then measured the expression levels of apoptotic pathway proteins. As depicted in (Figure 3E–I), TBHP exposure significantly enhanced the expression of cleaved caspase‐3 and BAX, suppressed Bcl‐2 expression, and elevated the BAX/Bcl‐2 ratio. Notably, LL pretreatment significantly reversed all these TBHP‐induced alterations.

### 3.5 EGR1 Downregulation Correlates With LL‐Mediated Protection Against TBHP‐Induced Cochlear Hair Cell Damage

**FIGURE 4:**
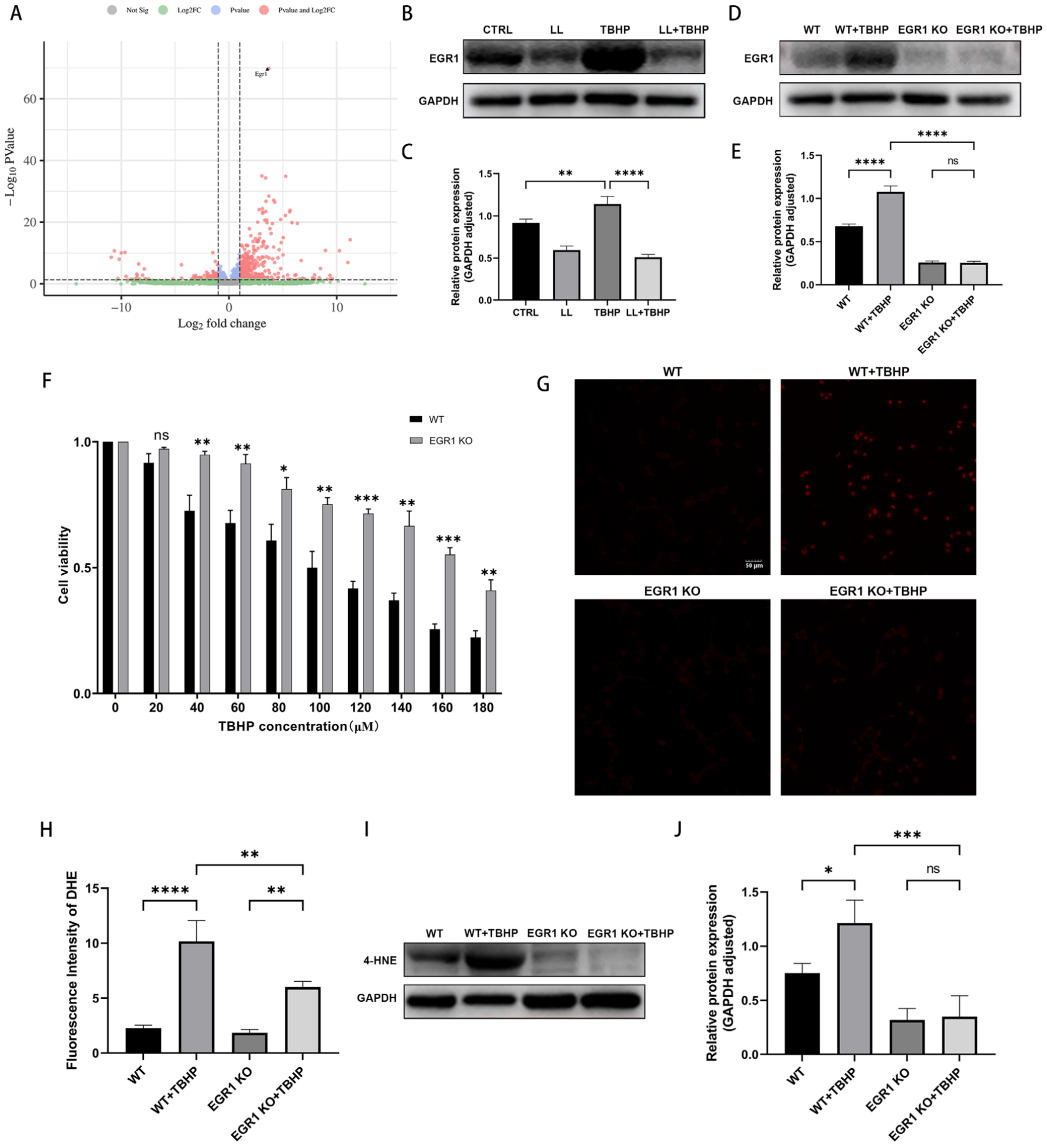
EGR1 gene knockout protects against TBHP‐induced reduction in cell viability and oxidative stress. (A) DEGs in cochlea post‐noise exposure (FDR < 0.05, |log₂fold change| > 1). (B, C) Western blot and quantitative analysis of EGR1 in HEI‐OC1 cells treated with LL and TBHP. (D, E) Western blot and quantitative analysis of EGR1 in WT and EGR1 KO cells. (F) Difference in cell viability of WT and EGR1 KO cells following TBHP treatment. (G, H) Representative fluorescence images and quantification of DHE‐stained WT and EGR1 KO cells following TBHP treatment. Scale bar = 50 μm. (I, J) Representative Western blot images and semiquantitative gray value analysis of 4‐HNE levels in WT and EGR1 KO cells treated with TBHP. *n* = 3 for all experiments. *p < 0.05; **p < 0.01; ***p < 0.001; ****p < 0.0001; ns = not significant.

To further investigate the molecular mechanisms underlying noise‐induced cochlear injury, transcriptome sequencing was performed on cochlear tissues at 1‐h post‐noise exposure. A total of 625 differentially expressed genes (DEGs) were identified, among which the expression of *Egr1* was significantly upregulated (Figure 4A). Western blot confirmed TBHP‐induced EGR1 upregulation in HEI‐OC1 cells, which was reversed by LL (Figure 4B,C).

To validate this observation, stable EGR1 knockout HEI‐OC1 (EGR1 KO) cells were generated using CRISPR/Cas9 technology. As presented in (Figure 4D,E), EGR1 protein expression was undetectable in EGR1 KO cells, and TBHP treatment failed to upregulate EGR1 in EGR1 KO cells. We then evaluated the sensitivity of EGR1 KO cells to TBHP: wild‐type (WT) and EGR1 KO cells were treated with the same concentration gradient of TBHP, and cell viability was measured. As shown in Figure 4F, when the TBHP concentration ranged from 40 to 180 μM, EGR1 KO cells exhibited significantly higher viability than WT cells.

**FIGURE 5:**
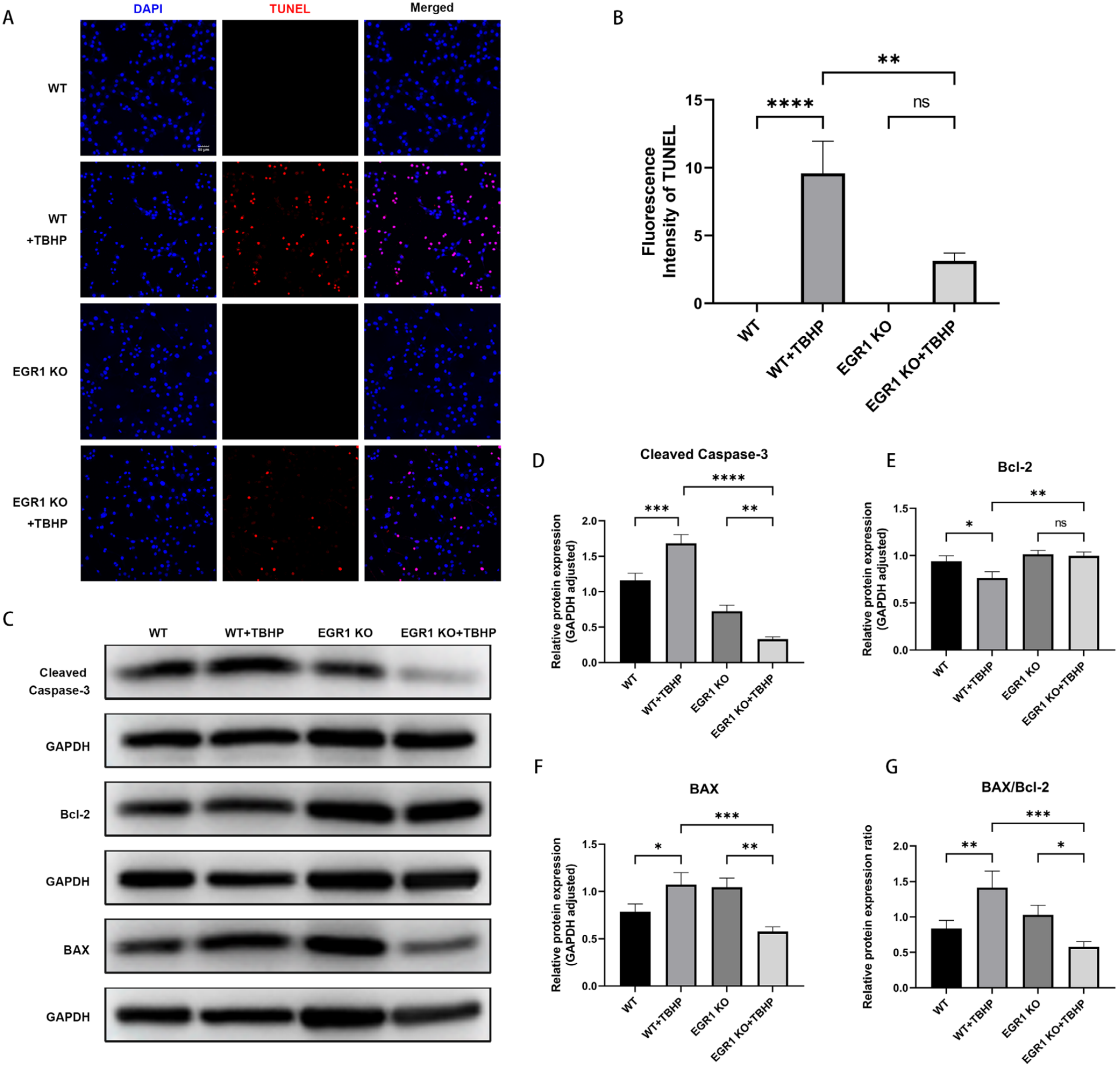
EGR1 gene knockout protects against TBHP‐induced cell apoptosis. (A, B) Representative fluorescence images of TUNEL and quantitative analysis of WT and EGR1 KO cells treated with TBHP. Scale bar = 50 μm. (C–F) Representative Western blot images showing the protein levels of cleaved caspase‐3, Bcl‐2, and BAX in WT and EGR1 KO cells treated with TBHP and the corresponding semiquantitative gray value analysis. (G) The relative expression ratio of BAX to Bcl‐2. *n* = 3 for all experiments. *p < 0.05; **p < 0.01; ***p < 0.001; ****p < 0.0001; ns = not significant.

Additionally, compared with WT cells, EGR1 KO cells did not exhibit a significant increase in DHE or 4‐HNE levels (Figure 4G–J). Meanwhile, the percentage of TUNEL‐positive cells was markedly lower in the EGR1 KO group than in the WT group (Figure 5A,B). Furthermore, after TBHP exposure, EGR1 KO cells had lower levels of cleaved caspase‐3, higher Bcl‐2 expression, and lower BAX expression than WT cells, which led to a lower BAX/Bcl‐2 ratio (Figure 5C–G).

### 3.6 The EGR1/SPRY4 Signaling Axis Is a Potential Mediator of LL‐Mediated *Protection Against TBHP‐Induced Cellular Damage*

**FIGURE 6:**
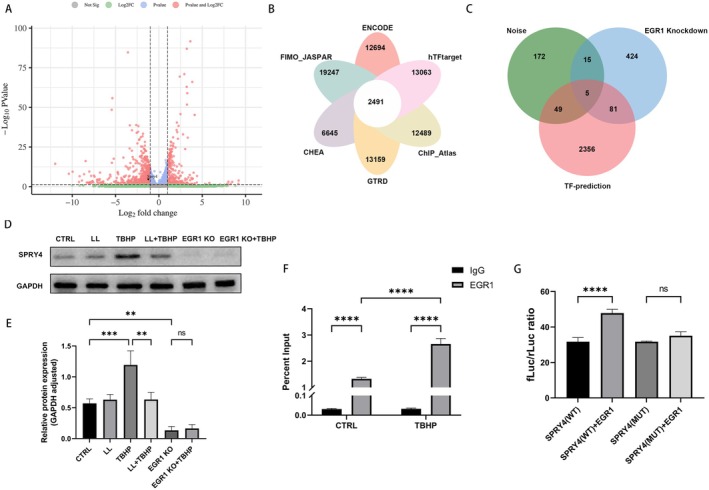
EGR1 regulates the transcription of SPRY4. (A) DEGs in the EGR1 KO cells (FDR < 0.05, |log_2_fold change| > 1). (B) Venn diagram of predicted EGR1 downstream target genes. (C) Intersection of noise‐induced upregulated genes, EGR1 KO downregulated genes, and predicted target genes. (D, E) SPRY4 Western blot and quantitative analysis in WT and EGR1KO cells treated with LL and TBHP. (F) ChIP‐PCR assay showing EGR1 binding to the SPRY4 promoter. (G) Dual‐luciferase reporter assay showing transcriptional activation of SPRY4 by EGR1. *n* = 3 for all experiments. **p < 0.01; ***p < 0.001; ****p < 0.0001; ns = not significant.

To further explore the function of EGR1, transcriptome sequencing was performed on EGR1 KO cells, leading to the identification of 952 DEGs (Figure 6A). RNA‐seq data confirmed that EGR1 was significantly downregulated in the EGR1 KO cells (log_2_fold change = −1.285, FDR = 2.88 × 10^−5^), confirming the successful and efficient knockout of EGR1. For the prediction of EGR1 downstream target genes, we conducted a screening using the web‐based tool TF‐Target Finder (https://jingle.shinyapps.io/TF_Target_Finder/) [39], and retrieved 2491 shared target genes from six transcription factor databases (Figure 6B). Subsequently, we took the intersection of three gene sets: the upregulated genes in noise‐exposed mouse cochleae, the downregulated genes in EGR1 knockout cells, and the predicted EGR1 target genes. This intersection analysis yielded five overlapping genes, namely *Spry4*, *Dusp6*, *Cebpb*, *Id1*, and *Zfp36l1* (Figure 6C). Among these candidates, *Cebpb*, *Id1*, and *Zfp36l1* are well‐characterized transcription factors. Thus, we hypothesized that *Spry4* acts as a downstream target gene of EGR1.

As shown in (Figure 6D,E), TBHP treatment significantly increased SPRY4 expression, whereas LL alleviated this TBHP‐induced upregulation. In EGR1 KO cells, SPRY4 expression was significantly reduced, but TBHP treatment failed to restore SPRY4 expression. ChIP‐PCR confirmed EGR1 binding to the SPRY4 promoter region, which can be enhanced by TBHP treatment (Figure 6F). Subsequently, Dual‐luciferase reporter assay revealed that EGR1 overexpression significantly elevated luciferase activity driven by the WT SPRY4 promoter, yet had no impact on the activity of the MUT SPRY4 promoter (Figure 6G).

## 4 Discussion

NIHL is a major public health problem lacking effective clinical treatments, highlighting the need for novel therapeutic strategies. This study represents the first demonstration that LL protects against NIHL by alleviating oxidative stress and suppressing apoptosis, which is accompanied by downregulation of the EGR1/SPRY4 axis. In vitro, EGR1 knockout mimics LL's protective effects, and EGR1 directly regulates SPRY4 transcription, suggesting that the EGR1/SPRY4 axis may contribute to LL's protective mechanism. Collectively, our findings identify LL as a promising candidate and the EGR1/SPRY4 axis as a potential therapeutic target for NIHL.

A core merit of this investigation is the employment of intratympanic LL administration, which delivers the drug directly to the cochlea, bypassing systemic toxicity and the blood‐labyrinth barrier. In vivo results showed that intratympanic LL significantly attenuated noise‐induced auditory threshold elevation and OHC loss in the treated ear. This delivery method has potential clinical translation value, as intratympanic injection is a well‐established procedure in otolaryngology.

The protective effect of LL against NIHL is mediated by mitigating oxidative stress and suppressing apoptosis. TBHP was selected as an in vitro model because it specifically induces ROS overproduction, which are the core pathological drivers of noise‐induced cochlear damage. LL dose‐dependently protected cochlear explants and HEI‐OC1 cells against TBHP‐induced injury, consistent with our in vivo findings of reduced OHC loss and auditory threshold elevation. Specifically, LL reduced intracellular ROS production, stabilized mitochondrial membrane potential, attenuated lipid peroxidation, and suppressed apoptotic pathways. These findings align with LL's known antioxidant and antiapoptotic properties in other tissues [40, 41], extending its protective role to the auditory system. Notably, LL protected cochlear auditory nerve fibers, suggesting protection against noise‐induced cochlear synaptopathy, which underlies hidden hearing loss [42]. It is important to acknowledge that while TBHP effectively recapitulates the oxidative stress component of noise‐induced injury, it does not fully replicate the mechanical damage to cochlear structures caused by intense noise. However, our combined in vivo and in vitro data strongly support that LL's protective effect is centered on mitigating oxidative stress, a key common pathway underlying both TBHP and noise‐induced damage.

A major novel finding of this study is the identification of the EGR1/SPRY4 axis as a potential regulatory pathway of LL's protective effect. EGR1 has been previously implicated in the regulation of oxidative stress [43, 44]. Consistently, our study confirmed that EGR1 expression is upregulated in TBHP‐treated HEI‐OC1 cells and this effect is reversed by LL, suggesting that LL may exert its otoprotective activity by regulating EGR1 expression. EGR1 knockout enhanced cell resistance to oxidative stress and apoptosis, suggesting a possible pro‐damaging role in NIHL. Mechanistically, ChIP‐PCR and dual‐luciferase reporter assays unambiguously verified that SPRY4 is a direct downstream target of EGR1: EGR1 binds directly to the SPRY4 promoter and transcriptionally regulates its expression, revealing a putative molecular link associated with NIHL.

Nevertheless, this study has several limitations. It remains unclear whether LL exerts its protective effects directly through the EGR1/SPRY4 signaling pathway. Moreover, the precise roles of EGR1 and SPRY4 in the pathogenesis of NIHL remain to be further validated in in vivo animal models.

## 5 Conclusion

Our results demonstrate that LL protects against noise‐induced auditory threshold elevation and OHC loss by alleviating oxidative stress and suppressing apoptosis. The EGR1/SPRY4 axis is likely involved in this protective process, as LL modulates EGR1/SPRY4 expression and EGR1 knockout enhances cellular stress resistance.

## Author Contributions

**Jia‐ning Guo:** investigation, writing – original draft, visualization, formal analysis. **Hong‐kai Mei:** investigation, data curation. **Rui Liang:** investigation, writing – review and editing, validation, supervision. **Peng‐wei Ma:** writing – review and editing, methodology, supervision. **Wei‐long Wang:** writing – review and editing, methodology, resources. **Jia‐wei Chen:** writing – review and editing, funding acquisition. **Zi Wang:** data curation, formal analysis. **Hao Yuan:** formal analysis. **Yu‐qiang Lun:** data curation. **Wei Gao:** writing – review and editing, methodology, supervision, resources. **Lian‐jun Lu:** conceptualization, resources, supervision, funding acquisition, project administration, writing – review and editing.

## Funding

This work was supported by the National Natural Science Foundation of China, 82273600, 82401350.

## Ethics Statement

All animal experimental procedures were approved by the Institutional Animal Care and Use Committee of the corresponding institution (SYXK‐2024‐003, approved on March 7, 2025).

## Conflicts of Interest

The authors declare no conflicts of interest.

## Supporting information


**Figure S1:** Representative Imagine of the Tympanic Membranes of Mice at Different Time Points Observed Under a Surgical Microscope. (A): Normal tympanic membrane of a mouse before intratympanic injection. (B): Puncture hole was made in the pars tensa of the tympanic membrane. The hole location is marked with a circle. (C): Tympanic membrane of a mouse one day after intratympanic injection. (D): Tympanic membrane of a mouse 14 days after intratympanic injection.
**Table S1:** The plasmid carried two promoter sequences of the Spry4 gene (WT, MUT) used in the Dual‐Luciferase Reporter Gene Assay.

## Data Availability

The data that support the findings of this study are available from the corresponding author upon reasonable request.
