# Supplementary material for: Luteolin Protects Against Noise‐Induced Hearing Loss via Mitigating Oxidative Stress and Apoptosis, With Potential Regulation of the EGR1/SPRY4 Axis
Source: CNS Neurosci Ther. 2026 May 1;32(5):e70906. doi: 10.1002/cns.70906 (PMC13135030; doi:10.1002/cns.70906)
Supplement: Supplementary file 1 — Figure S1: Representative Imagine of the Tympanic Membranes of Mice at Different Time Points Observed Under a Surgical Microscope. (A): Normal tympanic membrane of a mouse before intratympanic injection. (B): Puncture hole was made in the pars tensa of the tympanic membrane. The hole location is marked with a circle. (C): Tympanic membrane of a mouse one day after intratympanic injection. (D): Tympanic membrane of a mouse 14 days after intratympanic injection. Table S1: The plasmid carried two promoter sequences of the Spry4 gene (WT, MUT) used in the Dual‐Luciferase Reporter Gene Assay. [file CNS-32-e70906-s001.docx]

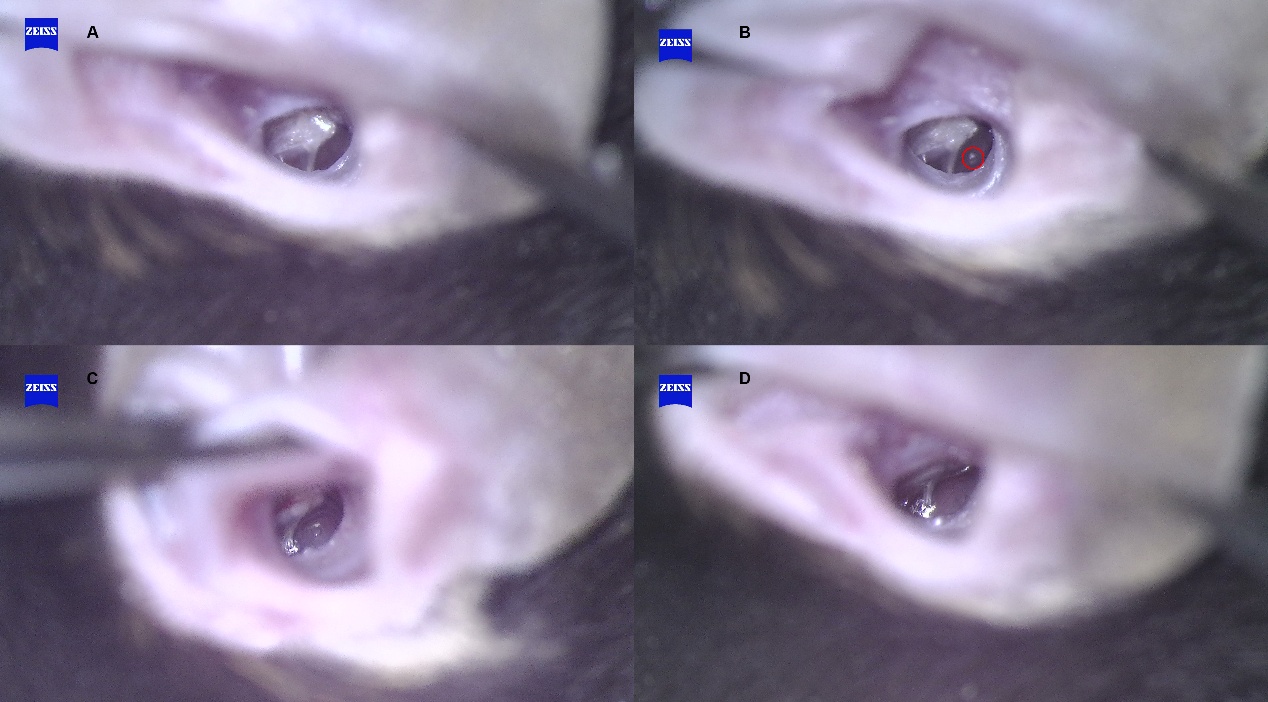


Supplementary Figure S1: Representative Imagine of the Tympanic Membranes of Mice at Different Time Points Observed Under a Surgical Microscope. (A): Normal tympanic membrane of a mouse before intratympanic injection. (B): Puncture hole was made in the pars tensa of the tympanic membrane. The hole location is marked with a circle. (C): Tympanic membrane of a mouse one day after intratympanic injection. (D): Tympanic membrane of a mouse 14 days after intratympanic injection.

| SPRY4-promoter-WT | TAGACTCAATAGGGAGAGAGACTTGGGCTGCAAGCCCTGAAATGCAGCATATTTACTTGTCCTTCGAAGGAAGTCCGAAAGGCAACCCCCCCCCCCCCGCCCCCTTTCTGGATGGAAGTTCTGTCCCAGGAAGGAAGCTGAAGCTCGTGGGAGTCTGTTCTGGTCCCAGGAACTGAGTAAGTAATCACTGAGACTAAGTCTGAGGGTTGCGTCCGCATTTTACGCTAGGACACGGTGACCTTGGGTTCTTGTCTGAACAGCCTGAGATGGAGCCGGAGCCCCAGCCCCAGGCCAGACCTCACCGGTGGGCTGTCTTTCAATAGGTTCCTCTGACAACAGCCGAGCGAGCACAGTCTTATGATGGCATAGGAAGAAAAAGTCTTACAACCTATGAAGGCCAAACACCAGCAAGATAGATCTATCTTGTTGAGGATTCAGACAGAGAACCAGTTCTTATATTGCATCAAAGCCATCTCTATCCCAGGCTTTCCGGTTGCTGGACGGAGCTGGGCCTGATGCTGGAGCGCCTGACTACAGGAGTCAGGCGGAATGGAGGGGCTTCTCTTTTCACCGGTTTGGGTTGCTACCCCCTAGCAAAGAAAGGCTCTCCGGCCATTTCCATTCTCTCTGTGGCTCAAGAGCCCTGTTATTTAATACCGTGGCCACCCTGGCAGCATCCTGCCGTGGCCTGCTCCACCCATCCACCTGGCTCATTCTCCCTATTCCTTTAGGGGAGTGAGGTGGGGGGAGCATTAGAGAAAACTGCCTTCTATAACAAAGGGGAGAGGGCCACAAGAATACAAAGGCCTGGAAGGACTGGAAGACAAGGACGCGAGTGGTGAAGGGGGCACCTAGCCAGGGCAGCAGAGGGCGGGCGGAAGCTCCAGGCACTGCGGACATTACACTGGGGCGGGGGAGAGGCGGGGCGCTGAGAGGTGAGGGCCAGGCCTCTGGCTGAGTGGACAGAATCGCCCCGGGGGGTACTAGAGCCGGAAGAGGCCTCGGCCCAGATACTGTCCACACCCAAGAAATGGGAGGAGTGGTCTGCACTTAGAAAGGGAGCCAGATTCACTCAATAACCCCCACCCTGCCCTTCTTTGAAATAGACAAAGGACATTTTCTTAATTTTTGGCAAACGTGGCTGGAAGGTCCCTGCCCCCGACGTCCCCAGGCGCCACAGATCTTGAAAAAGAGAAGGTCCCCAGTAGGGGTCCTCTCGCTCCCCCTCCCCATCGCCTGCGGTGACAAGAGATACGCAAATCGAGGTGTGTCCTCCCCCACCCTGCGCAGAGCCCAGGATCCGGAGGCCGCAGCGCCCCCCACCCTCTAGTCACACTGATGGTTCCTGCCCAAGCCCGCAGTGGCGTGAAGGTGGCCTTTACCTCCTCTCGCAGGCTGGGAGGAGGGTACAACCCACGCAGCTAAGCCAGCTGCTGCAGTCGCCGCCGCCCTCCATTCATTACAAAGAAAAAGGAAAAAAAAAAAAAAAAAGGAACAACAACCTCCACCCCTCTTTGTAAAAGAACGACGGCCCCAGGAGGAGCAACAGCCGGCCCCAGCAATCACGGGTTAAGCTCTTTTCCGGCCCCCCATTCATAAAAAACGGAGCCTAGCGCATGCGCGCACGGGGTCACTTGTGCCCACCCATTCATAGAATCGTGAGCGGCGCGCGTGCGCACTGGAGCTCTCTGCCCTCCCATTCATAAAAAAGCCATTTTCCCAGGCAAGGGTTGCAACATCGCCGCTGAGGCAGCGAACAGAGCTGACAGCGCGGAGCTGGCGCTGCAGGGCTCAGGGAGCTTTGCCGGCTCCTCCGACTGACGTCTGCGACTTCAACGGCGACTGACCCACTCGGGTTCGGGGATTTACACAGACGTGGAGCGATGCTTGTGACTCTGCAGCTCCTCAAAGTAAGTCTACAGCCGGTTTGGG |
| --- | --- |
| SPRY4-promoter-MUT | TAGACTCAATAGGGAGAGAGACTTGGGCTGCAAGCCCTGAAATGCAGCATATTTACTTGTCCTTCTGGTCACCAGATGAGGGACTTTTGGCCTCCACTTCTAGATCTGACAAATGGGAACAGTGATAAGTACAGAAGGAAGTCCGAAAGGCAACCCTGGATGGAAGTTCTGTCCCAGGAAGGAAGCTGAAGCTCGTGGGAGTCTGTTCTGGTCCCAGGAACTGAGTAAGTAATCACTGAGACTAAGTCTGAGGGTTGCGTCCGCATTTTACGCTAGGACACGGTGACCTTGGGTTCTTGTCTGAACAGCCTGAGATGGAGCCGGAGCCCCAGCCCCAGGCCAGACCTCACCGGTGGGCTGTCTTTCAATAGGTTCCTCTGACAACAGCCGAGCGAGCACAGTCTTATGATGGCATAGGAAGAAAAAGTCTTACAACCTATGAAGGCCAAACACCAGCAAGATAGATCTATCTTGTTGAGGATTCAGACAGAGAACCAGTTCTTATATTGCATCAAAGCCATCTCTATCCCAGGCTTTCCGGTTGCTGGACGGAGCTGGGCCTGATGCTGGAGCGCCTGACTACAGGAGTCAGGCGGAATGGAGGGGCTTCTCTTTTCACCGGTTTGGGTTGCTACCCCCTAGCAAAGAAAGGCTCTCCGGCCATTTCCATTCTCTCTGTGGCTCAAGAGCCCTGTTATTTAATACCGTGGCCACCCTGGCAGCATCCTGCCGTGGCCTGCTCCACCCATCCACCTGGCTCATTCTCCCTATTCCTTTAGGGGAGTGAGGTGGGGGGAGCATTAGAGAAAACTGCCTTCTATAACAAAGGGGAGAGGGCCACAAGAATACAAAGGCCTGGAAGGACTGGAAGACAAGGACGCGAGTGGTGAAGGGGGCACCTAGCCAGGGCAGCAGAGGGCGGGCGGAAGCTCCAGGCACTGCGGACATTACACTGGGGCGGGGGAGAGGCGGGGCGCTGAGAGGTGAGGGCCAGGCCTCTGGCTGAGTGGACAGAATCGCCCCGGGGGGTACTAGAGCCGGAAGAGGCCTCGGCCCAGATACTGTCCACACCCAAGAAATGGGAGGAGTGGTCTGCACTTAGAAAGGGAGCCAGATTCACTCAATAACCCCCACCCTGCCCTTCTTTGAAATAGACAAAGGACATTTTCTTAATTTTTGGCAAACGTGGCTGGAAGGTCCCTGCCCCCGACGTCCCCAGGCGCCACAGATCTTGAAAAAGAGAAGGTCCCCAGTAGGGGTCCTCTCGCTCCCCCTCCCCATCGCCTGCGGTGACAAGAGATACGCAAATCGAGGTGTGTCCTCCCCCACCCTGCGCAGAGCCCAGGATCCGGAGGCCGCAGCGCCCCCCACCCTCTAGTCACACTGATGGTTCCTGCCCAAGCCCGCAGTGGCGTGAAGGTGGCCTTTACCTCCTCTCGCAGGCTGGGAGGAGGGTACAACCCACGCAGCTAAGCCAGCTGCTGCAGTCGCCGCCGCCCTCCATTCATTACAAAGAAAAAGGAAAAAAAAAAAAAAAAAGGAACAACAACCTCCACCCCTCTTTGTAAAAGAACGACGGCCCCAGGAGGAGCAACAGCCGGCCCCAGCAATCACGGGTTAAGCTCTTTTCCGGCCCCCCATTCATAAAAAACGGAGCCTAGCGCATGCGCGCACGGGGTCACTTGTGCCCACCCATTCATAGAATCGTGAGCGGCGCGCGTGCGCACTGGAGCTCTCTGCCCTCCCATTCATAAAAAAGCCATTTTCCCAGGCAAGGGTTGCAACATCGCCGCTGAGGCAGCGAACAGAGCTGACAGCGCGGAGCTGGCGCTGCAGGGCTCAGGGAGCTTTGCCGGCTCCTCCGACTGACGTCTGCGACTTCAACGGCGACTGACCCACTCGGGTTCGGGGATTTACACAGACGTGGAGCGATGCTTGTGACTCTGCAGCTCCTCAAAGTAAGTCTACAGCCGGTTTGGG |

Supplementary Table 1: The plasmid carried two promoter sequences of the Spry4 gene (WT, MUT) used in the Dual-Luciferase Reporter Gene Assay.
